# Fundamental trends within falling match rates: Insights from the past decade of Canadian residency matching data

**DOI:** 10.36834/cmej.69289

**Published:** 2020-07-15

**Authors:** Andy G.X. Zeng, Connor T.A. Brenna, Silvio Ndoja

**Affiliations:** 1 University of Toronto, Ontario, Canada; 2 University of Western Ontario, Ontario, Canada

## Abstract

**Background:**

The number of unmatched Canadian Medical Graduates (CMGs) has risen dramatically over the last decade. To identify long-term solutions to this problem, an understanding of the factors contributing to these rising unmatched rates is critical.

**Methods:**

Using match and electives data from 2009-2019, we employed machine learning algorithms to identify three clusters of disciplines with distinct trends in match and electives behaviours. We assessed the relationships between unmatched rates, competitiveness, rates of parallel planning, and program selection practices at a discipline level.

**Results:**

Across Canada, growth in CMGs has outpaced growth in residency seats, narrowing the seat-to-applicant ratio. Yet not all disciplines have been affected equally: a subset of surgical disciplines experienced a consistent decline in residency seats over time. Applicants to these disciplines are also at disproportionate risk of becoming unmatched, and this is associated with lower rates of parallel planning as quantified through clinical electives and match applications. This, in turn, is associated with the program selection practices of these disciplines.

**Conclusion:**

Long-term solutions to the unmatched CMG crisis require more nuance than indiscriminately increasing residency seats and should consider cluster specific match ratios as well as regulations around clinical electives and program selection practices.

## Abstract (fr)

**Contexte:**

Le nombre de diplômés canadiens en médecine (DCM) non jumelés a augmenté considérablement au cours des dix dernières années. Afin de trouver des solutions à long terme à ce problème, il est primordial de comprendre les facteurs qui contribuent à cette hausse.

**Méthodes:**

À l’aide des données de jumelages et de stages à option de 2009 à 2019, nous nous sommes servis d’algorithmes d’apprentissage automatique afin d’identifier trois groupes de disciplines démontrant des tendances distinctes en ce qui a trait aux jumelages et au choix des stages à option. Nous avons évalué les relations entre le taux de diplômés non jumelés, la compétitivité, les taux de planification parallèle et les pratiques de sélection des programmes pour chacune de ses disciplines.

**Résultats:**

Partout au Canada, la croissance des DCM a dépassé la croissance du nombre de postes de résidence, réduisant ainsi le ratio postes-candidats. Cependant, les disciplines n’ont pas toutes été touchées de la même manière: un sous-ensemble de disciplines en chirurgie a connu, au fil du temps, un déclin continu en ce qui a trait aux postes de résidence offerts. Les candidats de ces disciplines sont aussi exposés à un risque démesuré de ne pas être jumelés et ceci est lié à la réduction des taux de planification parallèle tels que quantifiés par les stages à option cliniques et les demandes de jumelage. Ceci est, par conséquent, lié aux pratiques de sélection des programmes de ces disciplines.

**Conclusion:**

Les solutions à long terme de la crise touchant les DCM non jumelés requièrent plus de subtilités que le simple fait d’augmenter sans distinction le nombre de postes de résidence. Elles devraient également prendre en compte les ratios de jumelage propres aux groupes de disciplines ainsi que les règlements concernant les stages à option et les pratiques de sélection des programmes.

## Introduction

To practice medicine, Canadian medical graduates (CMGs) must match into a residency training program. This process is facilitated by the Canadian Residency Matching Service (CaRMS), which matches applicants to residency programs based on ranked lists of preferences submitted by both parties. However, some applicants do not match to any residency position. They are considered ‘unmatched’ and may choose to compete for unclaimed residency seats in the second iteration of the match or re-apply in the subsequent year. As they wait to re-apply, many defer graduation with their peers so they are able to undertake clinical electives while preparing for another CaRMS cycle. Some unmatched CMGs may leave clinical medicine altogether. Although a vast majority of unmatched students are competent and were ranked by residency programs,^1^^,^^2^ they continue to face stigma within the profession alongside financial debt and substantial personal stressors.^2^^,^^3^^,^^4^

From 2009 to 2018, the number of unmatched CMGs after the first iteration of the match doubled from 107 to 222 while the unmatched count after both iterations of the match increased from 25 to 123.^5^ This dramatic increase in unmatched CMGs became the subject of reports by both the Association of Faculties of Medicine of Canada (AFMC) and the Canadian Federation of Medical Students (CFMS), and has prompted government responses including the one-time addition of 53 supernumerary residency seats in Ontario.^1^^,^^6^^,^^7^ The benefits of these policies and others have been felt in the 2019 match, where there were 174 unmatched CMGs after first iteration and 62 unmatched after both iterations—a clear improvement from 2018. While these short-term successes are encouraging, long-term solutions have not yet materialized.^8^

Given the substantial implications for students and Canada’s future physician workforce, a deep understanding of the factors contributing to the rising unmatched rates is crucial for informing policies aimed at reducing the number of unmatched CMGs. In our present study, we conduct an in-depth analysis of publicly available CaRMS data from the past decade to identify factors associated with unmatched rates in the first iteration of the residency match. In particular, we identified three clusters of disciplines with distinct match outcomes, and demonstrate that this discrepancy is associated with residency seat allocation, applicant elective choices, and program selection practices.

## Methods

### Terminology clarifications

The terminology we use refers exclusively to the first iteration of the residency match. When we discuss applicants to any discipline, we are referring to CMGs who ranked that discipline as their first choice. *Unmatched* refers to applicants who applied to a specific discipline as their first choice but did not match to any residency position in the first iteration of the CaRMS match. *Diversity of electives* refers to how evenly distributed applicants’ electives are across clinical disciplines, as quantified through an approximation of Simpson’s Index of Diversity.^9^ We use diversity of electives and frequency of parallel applications to alternative disciplines to understand the extent to which CMGs engage in ‘parallel planning’ to prepare to apply for more than one residency discipline. *Alternative Outcomes* refers to the outcomes for applicants who did not match into their first-choice discipline: the probability that applicants will match into an alternative discipline as opposed to becoming unmatched.

### Data

We obtained publicly available residency match data spanning 2009-2019 and electives data spanning 2013-2019 from CaRMS.^5^ We focused our analysis entirely on data from the first iteration of the residency match, as a limited number of disciplines has unfilled seats available in second iteration. We excluded direct-entry clinician-scientist tracks from our analysis owing to the absence of dedicated electives in those disciplines, and thus a lack of electives data.

### Hierarchical clustering

We calculated 11 primary summary statistics summarizing match and electives behaviour for each discipline in each year (Table 1). For each discipline, we took the median of each statistic from 2013 (the earliest available time point for electives data) to 2019 and performed complete linkage hierarchical clustering using the correlation-based distance between disciplines.^10^ We determined the optimal number of clusters (*k*) to be three through the elbow method combined with visual inspection of the clustering dendrogram.^10^ To visualize clustering results, we employed t-SNE,^11^ a machine learning approach that visualizes discipline similarity across all 11 summary statistics in a 2-dimensional plot.

**Table 1:** Primary and composite statistics on the residency match

Metric	Description
***Primary Statistics (used for clustering)***	
Competitiveness	This is the ratio of applicants who ranked a discipline as their first choice over the number of seats available in that discipline
Proportion Unmatched	This is the proportion of applicants who ranked a discipline as their first choice that subsequently went unmatched in first iteration
Frequency of Parallel Applications	This is the proportion of applicants who ranked a discipline as their first choice that also ranked any other discipline on their application
Mean Electives within Matched Discipline	Amongst matched applicants, this is the mean number of distinct electives they have completed within the discipline they matched to
Mean Electives outside Matched Discipline	Amongst matched applicants, this is the mean number of distinct electives they have completed outside the discipline they matched to
Mean Other Disciplines with Completed Electives	Amongst matched applicants, this is the mean number of other disciplines that they completed electives in
Proportion Ranked with Discipline Elective	Amongst ranked applicants, this is the proportion who completed at least one elective in the discipline that they were ranked by
Proportion Matched with Discipline Elective	Amongst matched applicants, this is the proportion who completed at least one elective in the discipline that they were matched to
Proportion Matched with ≥ 3 Discipline Electives	Amongst ranked applicants, this is the proportion who completed at least three electives in the discipline that they were ranked by
Proportion Ranked with Program Elective	Amongst ranked applicants in a given discipline, this is the proportion who completed an on-site elective with the program they were ranked by
Proportion Matched with Program Elective	Amongst matched applicants in a given discipline, this is the proportion who completed an on-site elective with the program they matched to
***Composite Statistics***	
Diversity of Electives	Approximation of Simpson's Diversity Index,^6^ taking into consideration the number of distinct disciplines that electives were completed in and how distributed electives were across disciplines.
Alternative Outcomes (Matched to Alternative vs Unmatched)	Conditional probability of matching into an alternative discipline given that an applicant does not match to their first-choice discipline

### Machine learning models for identifying factors influencing unmatched rates

To identify factors influencing unmatched rates we constructed both a linear model (LASSO regression) and non-linear model (random forest regression) predicting unmatched rates from the 11 match and electives statistics. Briefly, we built both models using the ‘scikit-learn’ package in Python. We trained both models on 70% of the observations (*n*= 142) and tested them on a 30% hold-out set of 61 observations. LASSO regression was performed with leave-one-out cross validation. Hyperparameters for the random forest regressor were selected through a grid search to identify the optimal number of estimators and the maximum depth of each tree. After the models were built, we used the ‘shap’ package to determine the importance of each factor to each model in predicting unmatched rates.

### Analysis code

Cleaned residency match data used for the analysis, as well as all *R* and Python code used to perform clustering, model creation, and figure generation, are publicly available on GitHub at https://github.com/andygxzeng/carms_paper. We also provide the match and electives statistics from our analysis, summarised and visualized for each discipline, as a resource for medical students on the CFMS website: https://www.cfms.org/what-we-do/education/matchstats.

## Results

### CMG growth has outpaced growth in residency seats

From 2009 to 2019, the total number of residency seats increased by 17% from 2573 to 3020, with 89% of this growth taking place between 2009-2014 followed by a plateau. During this time, the number of CMGs increased by 28% at a steady rate from 2296 to 2934, resulting in a declining seat-to-applicant ratio from 1.13 seats per applicant in 2009 down to 1.03 seats per applicant in 2019.

### Residency disciplines cluster into three groups with distinct match behaviour

**Figure 1:**
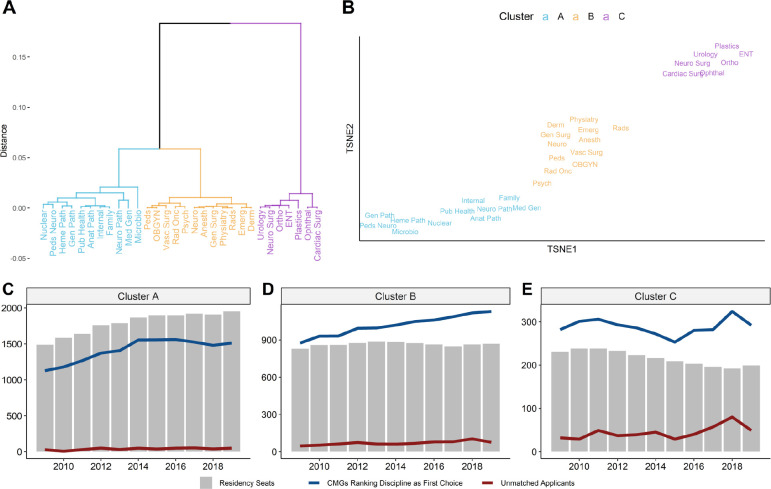
Hierarchical clustering of disciplines reveals distinct trends in applicant-to-seat ratios *A) Dendrogram depicting hierarchical clustering of match disciplines across 11 match and electives statistics from 2013-2019. The height/distance of branches between two disciplines depicts the extent of their differences across the 11 metrics. Disciplines are coloured by designated cluster. B) t-stochastic neighbour embedding (TSNE) plot providing a 2D visualization of discipline to discipline similarity across 11 match and electives statistics. Disciplines that are closer together on the TSNE plot share greater similarity in their match and electives behaviour across the 11 metrics used. C-E) First iteration counts of Residency Seats (grey), Canadian Medical Graduate (CMG) Applicants (blue), and Unmatched Applicants (red) from 2009 to 2019. C) Aggregate first iteration counts for Cluster A specialties from 2009 to 2019. D) Aggregate first iteration counts for Cluster B specialties from 2009 to 2019. E) Aggregate first iteration counts for Cluster C specialties from 2009 to 2019*.

Through hierarchical clustering based on 11 primary summary statistics (Table 1), we identified three clusters of disciplines that each exhibit distinct patterns of match and electives behaviour (Figure 1A). We refer to these clusters as Cluster A, Cluster B, and Cluster C, and show the disciplines belonging to each cluster in Table 2. In Figure 1B, we provide a visualization of the similarity between disciplines across all 11 match and electives statistics, such that disciplines positioned closer to one another on the plot share greater similarity in electives behaviour and match outcomes. The disciplines and their relative values of each summary statistic are also depicted in a heatmap in Supplementary Figure S1.

Supplemental figures

**Table 2:** Distribution of specialties among the clusters

Cluster A		Cluster B		Cluster C	
**Discipline**	**Abbreviation**	**Discipline**	**Abbreviation**	**Discipline**	**Abbreviation**
Anatomical Pathology	Anat Path	Anesthesiology	Anesth	Cardiac Surgery	Cardiac Surg
Family Medicine	Family	Dermatology	Derm	Otolaryngology	ENT
General Pathology	Gen Path	Emergency Medicine	Emerg	Neurosurgery	Neuro Surg
Hematological Pathology	Heme Path	General Surgery	Gen Surg	Ophthalmology	Ophthal
Internal Medicine	Internal	Neurology	Neuro	Orthopedic Surgery	Ortho
Medical Genetics	Med Gen	Obstetrics & Gynecology	OBGYN	Plastic Surgery	Plastics
Medical Microbiology	Microbio	Pediatrics	Peds	Urology	Urology
Neuropathology	Neuro Path	Physical Medicine & Rehabilitation	Physiatry		
Nuclear Medicine	Nuclear				
Pediatric Neurology	Peds Neuro	Psychiatry	Psych		
Public Health & Preventive Medicine	Pub Health	Radiation Oncology	Rad Onc		
		Diagnostic Radiology	Rads		
		Vascular Surgery	Vasc Surg		

Re-examination of the changes in residency seats and CMG applicants from 2009 to 2019 reveals distinct patterns in seat-to-applicant ratios across the three clusters. During this period, Cluster A disciplines saw a growth in residency seats outpacing the growth in applicants (Figure 1C: applicants from 1127 to 1512 and seats from 1489 to 1952). In Cluster B disciplines, the number of residency seats stayed relatively constant while the number of applicants has increased, resulting in a steady increase in competition (Figure 1D: applicants from 875 to 1130 and seats from 829 to 869). In contrast, the number of residency seats in Cluster C disciplines has been consistently declining since 2011 while the number of applicants, though variable, has been growing in recent years (Figure 1E: applicants from 282 to 292 and seats from 231 to 199).

### Cluster C has disproportionately higher unmatched rates

**Figure 2:**
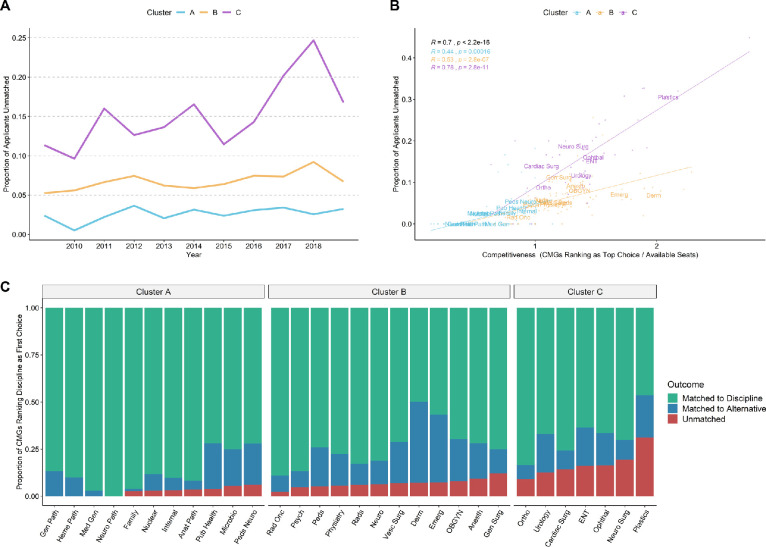
Cluster C disciplines have disproportionately high unmatched rates *A) Average proportion of CMG applicants unmatched in first iteration from 2009 to 2019, separated by cluster. B) Scatterplot depicting the relationship between competitiveness (applicant to seat ratio) and proportion of applicants unmatched in first iteration. Each point depicts competitiveness and unmatched rate per year for a discipline between 2013 - 2019. Text labels with discipline abbreviations are positioned at the average value for each discipline. Pearson correlation for all data points as well as for each cluster are portrayed with corresponding p-values. Linear regression trend lines for each cluster are also depicted. C) Stacked bar plot representing 2013 - 2019 averages of first iteration match outcomes of CMGs applying to a given discipline as their first choice, separated by cluster*.

We observed stark differences in unmatched rates across the three clusters from 2009 to 2019 (Figure 2A). Cluster A disciplines as a whole have a low unmatched rate (< 5%) with minimal increases over time (+0.7% every 5 years). Cluster B disciplines have a higher cluster-wide unmatched rate (5-10%) with modest increases over time (+1.1% every 5 years). In contrast, Cluster C disciplines have the highest unmatched rate (10-25%) and experienced more dramatic increases in unmatched CMGs over time (+4.6% every 5 years).

One obvious contributing factor to an applicant’s risk of going unmatched is the ‘competitiveness’ of the discipline they are applying to, represented as the number of applicants divided by the number of residency seats available in that discipline. While there is a strong global correlation between discipline competitiveness and unmatched rate (Figure 2B: *R* = 0.70, *p* < 2.2e-16), re-examination of this relationship by cluster reveals that this correlation is strongest and steepest within Cluster C disciplines (*R* = 0.78, *p* = 2.8e-11). This suggests that applicants to Cluster C disciplines face a disproportionately higher risk of becoming unmatched compared to those applying to equally competitive Cluster B disciplines. This is also reflected in Figure 2C, which depicts match outcomes of CMGs applicants to each discipline.

The finding of disproportionately higher unmatched rates in Cluster C disciplines than in equally competitive Cluster B disciplines suggests that the ratio of applicants to seats alone cannot explain these differences in unmatched rates. To identify other influencing factors, we built two machine learning models to predict unmatched rates from the 11 match and electives statistics and tracked which factors the models relied on the most in predicting unmatched rates (Supplementary Figure S2). While competitiveness remained the strongest predictor of unmatched probability, the number of electives that applicants completed in their first-choice discipline was the second most important predictor in both models, suggesting that electives behaviour may influence graduates’ risk of going unmatched.

### Case Study: Dermatology and plastic surgery

**Figure 3:**
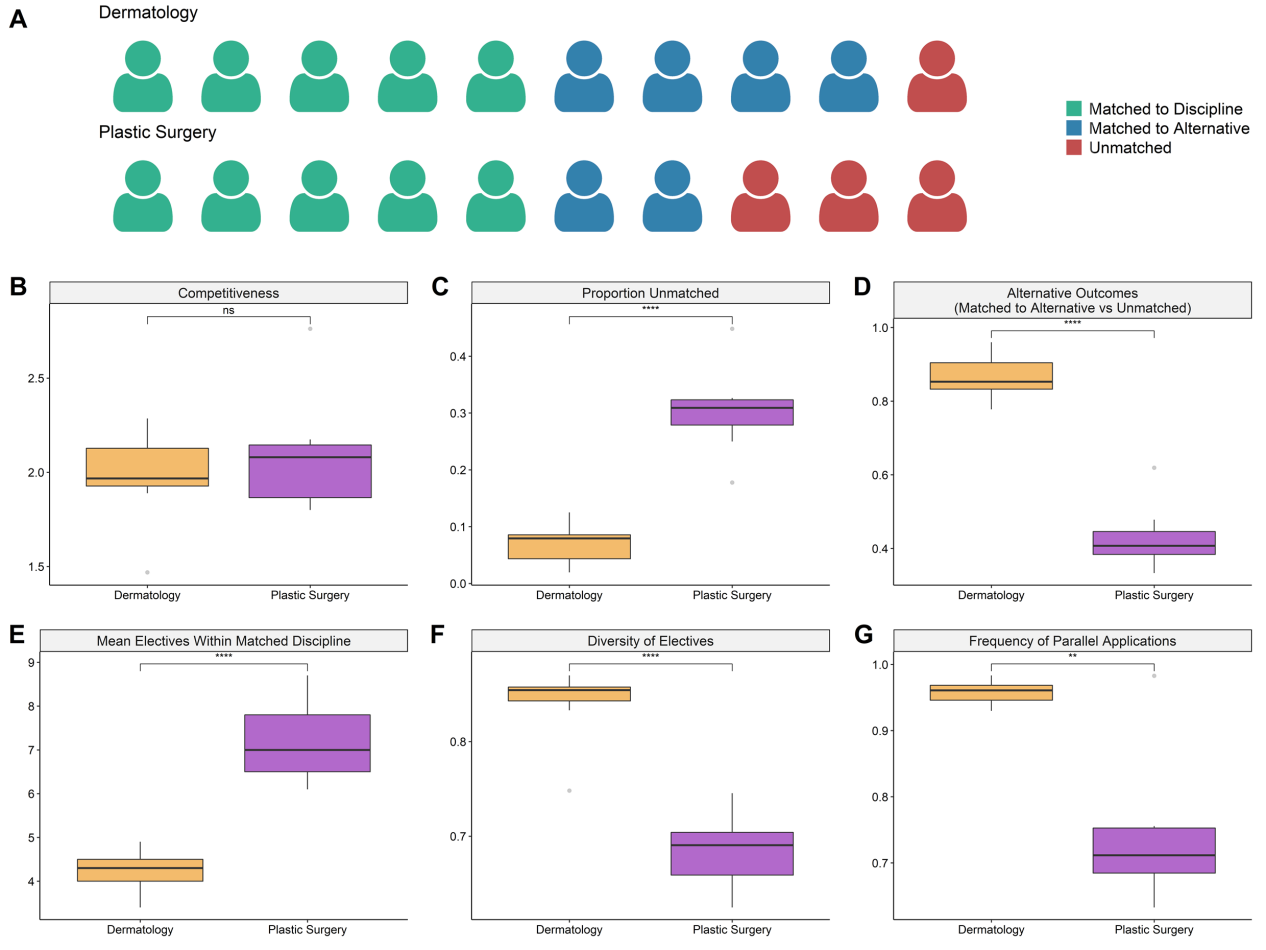
Case study of dermatology and plastic surgery reveals disproportionately higher unmatched rates and lower rates of parallel planning among Plastic Surgery applicants A) Modified waffle plot depicting relative first iteration match outcomes for CMGs who ranked Dermatology and Plastic Surgery as their first discipline. B-G) Boxplots comparing match and electives metrics for Dermatology and Plastic Surgery from 2013 - 2019. P-values were obtained through two-sided t-tests. ns=not significant; * p < 0.05; ** p < 0.01; *** p < 0.001; **** p < 0.0001. B-D) Depictions of discipline competitiveness, proportion of applicants unmatched in first iteration, and the probability of being unmatched for applicants who did not match to their first discipline. E-G) Depictions of the mean number of electives that CMGs who matched reported completing, the diversity of their electives across disciplines, and the proportion of applicants who submitted parallel applications to other disciplines.

To gain insight into factors influencing the disproportionately high unmatched rate observed in Cluster C, we compared the match and electives behaviour of the two most competitive disciplines: dermatology (Cluster B) and plastic surgery (Cluster C). Dermatology and plastic surgery are equally competitive, with approximately two times the number of applicants than available residency seats (Figure 3B), yet the unmatched rate for plastic surgery is more than three times higher than for dermatology (Figure 3C: 31% vs 7%). Notably, an examination of alternative outcomes revealed that CMGs who applied to dermatology as their first choice but did not successfully match to dermatology were very likely to match into an alternative discipline, while those who applied to plastic surgery as their first choice but did not successfully match to plastic surgery were more likely to go entirely unmatched (Figure 3A and 3D: 87% vs 43%).

We observed substantial differences in electives and match application behaviour between applicants to plastic surgery and applicants to dermatology. CMGs who matched into plastic surgery reported completing significantly more distinct electives in the discipline they matched to than those in Dermatology (Figure 3E: 7 vs 4) and displayed a significantly lower diversity of electives across disciplines (Figure 3F). Furthermore, a lower proportion of applicants to plastic surgery submitted parallel applications to other disciplines than those in dermatology (Figure 3G: 74% vs 96%). Taken together, the reduced electives diversity and relative disparity of parallel applications suggests lower rates of parallel planning among plastic surgery applicants in comparison with dermatology applicants.

### Lower rates of parallel planning correlate with poor alternative outcomes

**Figure 4:**
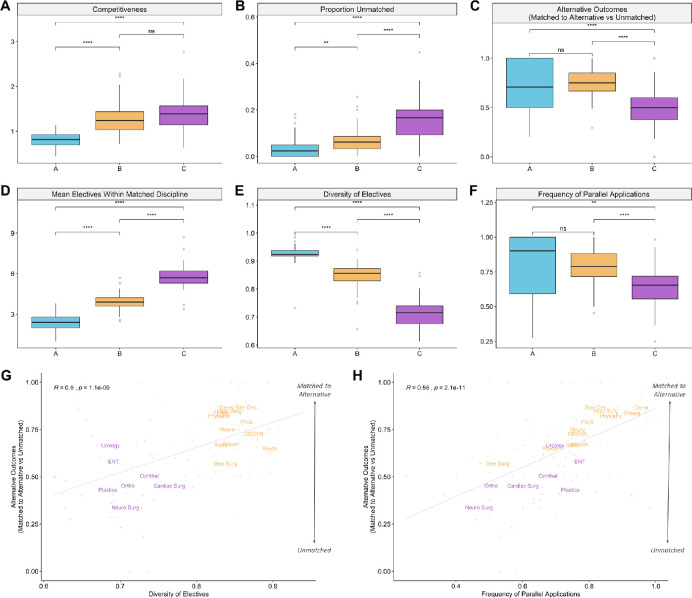
Lower rates of parallel planning among Cluster C disciplines correlate with poor alternative outcomes A-F) Boxplots comparing match and electives metrics across clusters from 2013 - 2019. P-values were obtained through two-sided t-tests. ns=not significant; * p < 0.05; ** p < 0.01; *** p < 0.001; **** p < 0.0001. A-C) Depictions of discipline competitiveness, proportion of applicants unmatched in first iteration, and the probability of being unmatched for applicants who did not match to their first choice discipline. D-F) Depictions of the mean number of electives that CMGs who matched to that discipline reported completing, the diversity of their electives across disciplines, and the proportion of applicants to that discipline who submitted parallel applications to other disciplines. G-H) Scatterplots depicting the probability of being unmatched for Cluster B and Cluster C applicants who did not match to their first choice discipline and its relationship to diversity of electives and proportion with parallel applications. Each point depicts competitiveness and unmatched rate per year for a discipline between 2013 - 2019. Text labels with discipline abbreviations are positioned at the average value for each discipline.

This trend of disproportionately high unmatched rates together with low rates of parallel planning also holds for Cluster C disciplines in aggregate. Cluster B and Cluster C disciplines do not differ significantly in competitiveness, with applicant to seat ratios of 1.3 and 1.4 respectively (Figure 4A), yet Cluster C disciplines have significantly higher unmatched rates (Figure 4B). Critically, alternative outcomes of Cluster C applicants are also poor: those who do not match into their first-choice discipline have only a 49% chance of matching into an alternative discipline, in contrast with Cluster A or Cluster B disciplines where unsuccessful applicants have a 75% chance of matching into an alternative (Figure 4C). CMGs applying to Cluster C disciplines show less diversity in their choice of electives (Figure 4E) and do not submit parallel applications to other disciplines as frequently (Figure 4F) as their Cluster B counterparts.

Among Cluster B and Cluster C disciplines, where the number of applicants generally exceeds the number of residency seats, both elective diversity and frequency of parallel applications were significant determinants of favourable alternative outcomes. In particular, disciplines where unsuccessful applicants were frequently able to match into alternatives tended to be ones where applicants had higher electives diversity (Figure 4G: *R* = 0.5, *p* = 1.1e-9) and a higher frequency of parallel applications (Figure 4H: *R* = 0.55, *p* = 2.1e-11).

### Residency program selection practices correlate with electives diversity

**Figure 5:**
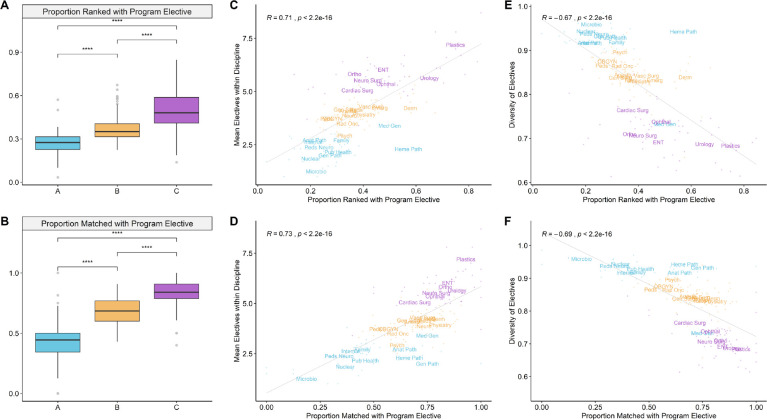
Residency program selection practices correlate with electives diversity A-B) Boxplots comparing program selection practices across clusters from 2013 - 2019. P-values were obtained through two-sided t-tests. ns=not significant; * p < 0.05; ** p < 0.01; *** p < 0.001; **** p < 0.0001. Metrics depicted are A) proportion of ranked applicants who completed on-site electives in the program they were ranked by and B) proportion of matched applicants who completed on-site electives in the program they matched to. C-F) Scatterplots depicting the relationship between electives and program selection practices. Each point depicts competitiveness and unmatched rate for a discipline in any given year between 2013 - 2019. Text labels with discipline abbreviations are positioned at the average value for each discipline. C-D) The number of electives that applicants complete in a discipline as it relates to the frequency of completed on-site program electives among ranked or matched applicants in that discipline. E-F) The electives diversity of applicants as it relates to the frequency of completed on-site program electives among ranked or matched applicants.

We next examined the importance of on-site program electives in applicant selection practices across the three clusters. We used two summary statistics that capture this behaviour: the proportion of applicants who completed an elective with the program they were ranked by, and the proportion of matched applicants who completed an elective with the program they were matched to. The former solely reflects selection practices of the program while the latter reflects preferences of both the program and the applicants matched to it (Table 1). We find that Cluster C disciplines show a significantly stronger preference for applicants with on-site program electives (Figures 5A-5B). Among disciplines with strong preferences for on-site program electives, successful applicants tended to complete more electives in their matched discipline (Figures 5C-5D) and had an associated reduction in the overall diversity of their electives (Figures 5E-5F).

## Discussion

In our study, we documented the recent decline in the overall ratio of residency seats to CMGs arising from unbalanced rates of growth between CMG applicants and residency seats. One reason for this discrepancy is that annual numbers of residency seats and medical school graduates may be determined by different governing bodies (e.g. in Ontario, residency seats are funded through the Ministry of Health while medical school seats are funded through the Ministry of Education). Broadly, the asymmetric growth of medical student spots over residency spots has increased the competitiveness of the match. At a discipline level, this change is a metric that is highly correlated with unmatched rates. This highlights the importance of interventions aimed at harmonizing residency seat allocation with medical school enrolment.

Through hierarchical clustering analysis we show that not all disciplines are affected equally by these declining match ratios. In particular, we identify groups of disciplines that are minimally affected (Cluster A), moderately affected (Cluster B), and severely affected (Cluster C). Cluster A specialties typically have many more seats than applicants, allowing for generally favorable match outcomes, with an unmatched rate of <5% among Cluster A applicants. Cluster B and Cluster C disciplines have similar applicant to seat ratios, yet Cluster C disciplines have a significantly higher rate of unmatched applicants. We propose that this difference can in part be explained by lower rates of parallel planning among applicants to Cluster C disciplines.

Notably, we also observe stark differences in alternative outcomes: applicants to Cluster C disciplines who did not match to their first-choice discipline were less likely to match to an alternative and more likely to go entirely unmatched in comparison to their Cluster B counterparts. In disciplines where CMG applicants typically outnumbered residency seats, these alternative outcomes are significantly correlated with rates of parallel planning which are lower among Cluster C disciplines. Together, this suggests that the disproportionately high unmatched rates among Cluster C disciplines may be in part explained by lower rates of parallel planning among applicants. Accordingly, it may be feasible that, in the absence of sufficient parallel planning, the unmatched rates within these disciplines could be more sensitive to changes in seat-to-applicant ratios compared to other disciplines with higher rates of parallel planning.

In considering factors that may contribute to the lower rates of parallel planning among applicants to Cluster C disciplines, we suggest three possible explanations that need to be investigated. First, applicants to Cluster C disciplines may be less interested in considering alternative disciplines. Second, skills and experiences specific to Cluster C disciplines may be less applicable to other disciplines in Cluster A or Cluster B, leading to a lack of clear alternatives to plan a parallel path. Third, applicants to Cluster C disciplines may feel compelled to maximize their discipline-specific electives based on their perceptions of program selection practices, such as prerequisite numbers of discipline-specific reference letters or preferences for on-site program electives. We were partially able to evaluate the last possibility by examining, among all the applicants who were ranked by or matched to a program, how many had completed on-site electives with that program. Indeed, more applicants who were ranked by or matched to Custer C programs had completed on-site electives with those specific programs, compared to the similarly competitive Cluster B disciplines. This is supported by a study demonstrating that plastic surgery programs explicitly favour on-site electives when ranking applicants^12^ and is reflected in growing concerns that electives are being approached as serial auditions rather than opportunities for exploration.^13^ Given the emphasis placed on on-site electives among Cluster C disciplines, parallel planning may actually jeopardize applicant success in matching to these disciplines and this may contribute to their disproportionately high unmatched rate in comparison to their Cluster B counterparts.

At face value, these findings contradict a recent study which reported no relationship between electives planning and match success among applicants from two years of data within one institution.^14^ However, the Courneya study examines overall match outcomes of applicants applying to what they define as “High Demand / Low Supply” disciplines, which excludes Internal Medicine and Family Medicine yet includes other Cluster A and Cluster B disciplines where supply outnumbers demand. When we examine this relationship at the level of individual disciplines, we find a striking correlation between the mean number of electives completed within any particular discipline and the discipline-wide unmatched rate (*R* = 0.70, *p* < 2.2e-16, 2015-2019) and have demonstrated how electives correspond to disproportionately high unmatched rates when grouping disciplines by cluster. Additionally, we find that the association between number of electives completed and unmatched rates are strongest amongst the top third most competitive disciplines, and this is also the case for the negative association between parallel applications and unmatched rates (Supplementary Figure S3). We have demonstrated that there is indeed a difference in match rates between those that do and do not “parallel plan” when applying for Cluster C specialties, a granularity not previously reported in the literature.

In 2018, the AFMC introduced their Electives Diversification policy, which will set a nation-wide cap of 8 weeks of electives in any direct-entry discipline. Given a 2-week minimum elective length, this amounts to a maximum of 4 distinct electives in any discipline. This policy will strongly affect Cluster C applicants who report completing an average of 6.0 distinct electives (5-year average, SD of 0.9), moderately affect Cluster B applicants (5-year average of 4.0, SD of 0.6), and minimally affect Cluster A applicants (5-year average of 2.2, SD of 0.7). Enforcing higher electives diversity among Cluster C applicants will likely improve alternative outcomes, increasing the likelihood that unsuccessful applicants to those disciplines may match into an alternative specialty as opposed to going unmatched. However, it remains to be seen what comes of this policy as it does not address program selection practices^15^ and applicant behaviours which contribute to the disproportionately high unmatched rates among Cluster C disciplines.

Our study utilizes publicly available data from CaRMS, and as such we are limited to making conclusions about associations at a discipline level and cannot comment directly on individual or program-related factors associated with unmatched rates. An additional limitation is that the electives data were collected from information that students self-reported on their applications and may thus be subject to variation arising from different interpretations among applicants regarding which experiences qualify as clinical electives.

For future studies, it will be important to examine physician workforce planning and how residency seats and medical school enrolment can best be harmonized on a provincial basis. Additionally, qualitative analyses of how perceived program selection practices influence applicant electives and career planning choices may offer strategies to improve rates of parallel planning among CMGs. We hope that the insights from this analysis will be useful for informing career advising across Canadian medical schools as well as future policy decisions aimed at reducing the number of unmatched CMGs.
