# Supplementary material for: Fundamental trends within falling match rates: Insights from the past decade of Canadian residency matching data
Source: Can Med Educ J. 2020 Jul 15;11(3):e31–42. doi: 10.36834/cmej.69289 (PMC7378143; doi:10.36834/cmej.69289)
Supplement: Supplementary file 1 — Supplemental figures [file CMEJ-11-e031-s001.pdf]

Supplemental figures

Figure S1: Heatmap of cluster features across 11 match and electives statistics

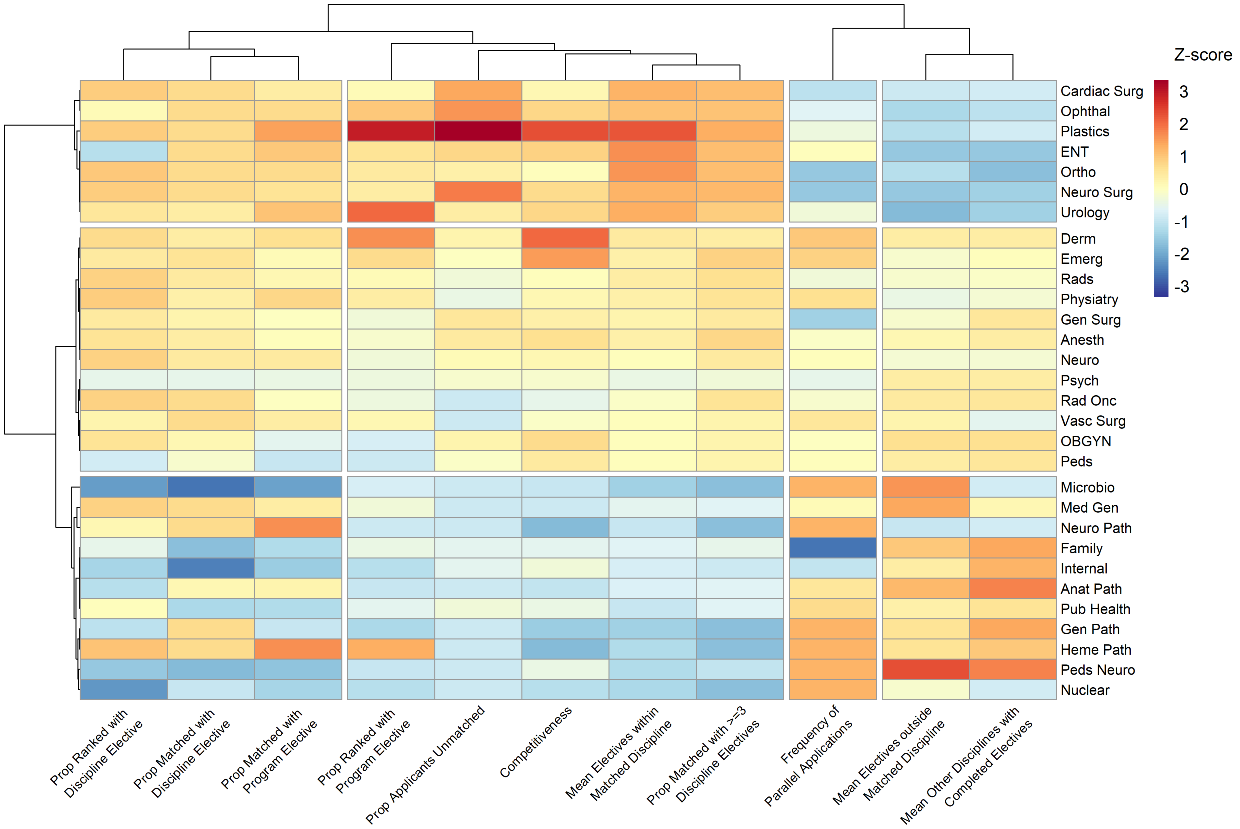

Heatmap depicting Z-score of each match and electives statistic for each discipline, using the median from 2013-2019. Columns depict match statistics and rows depict residency match disciplines, arranged by hierarchical clustering.

Figure S2: Machine learning models of unmatched rates highlight competitiveness and electives as key predictors of discipline-level unmatched rates

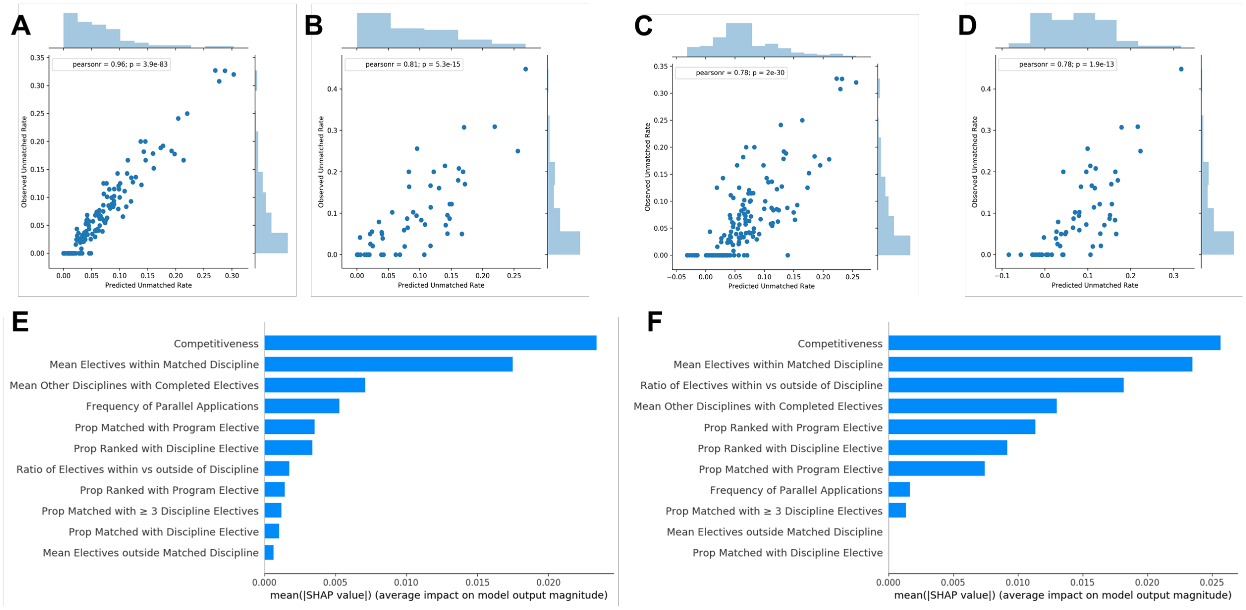

A-D) Correlation plots depicting the relationship between observed unmatched rates for match disciplines from 2013 – 2019 and the predicted unmatched rates from one of two machine learning models. Histograms on the margins depict the distribution of observed and predicted unmatched rates. A-B) Predictions from a random forest regressor model on the training set of 142 observations (A) and the validation set of 61 observations (B). C-D) Predictions from a LASSO regression model on the training set of 142 observations (C) and the validation set of 61 observations (D). E-F) SHAP values depicted for each of the 11 variables in the two machine learning models. Variables are ordered from most important to least important, and their SHAP value depicts the strength of their impact on model predictions. E) SHAP values for the random forest regression model. F) SHAP values for the LASSO regression model.

Figure S3: Electives and parallel applications are more strongly associated with unmatched rates at high competition levels

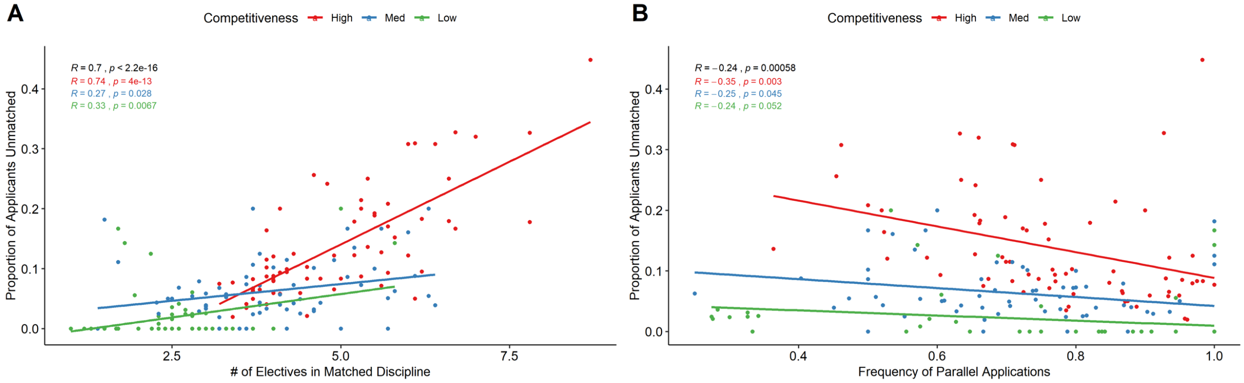

A) Relationship between the number of electives that applicants complete within a discipline and the unmatched rate for that discipline, across different levels of competitiveness (split evenly three-ways). B) Relationship between the frequency of parallel applications for a discipline and the unmatched rate for that discipline, across different levels of competitiveness.
